# Assessment of Cardiotoxicity after a Single Dose of Combretastatin A4-Phosphate in Dogs Using Two-Dimensional Speckle-Tracking Echocardiography

**DOI:** 10.3390/ani12213005

**Published:** 2022-11-02

**Authors:** Gitte Mampaey, Arnaut Hellemans, Hilde de Rooster, Tom Schipper, Eline Abma, Bart J. G. Broeckx, Sylvie Daminet, Pascale Smets

**Affiliations:** 1 Small Animal Department, Faculty of Veterinary Medicine, Ghent University, 9820 Merelbeke, Belgium; 2 Department of Veterinary and Biosciences, Faculty of Veterinary Medicine, Ghent University, 9820 Merelbeke, Belgium

**Keywords:** canine, systolic dysfunction, myocardial damage, heart failure, cardiac troponin I, global longitudinal strain

## Abstract

**Simple Summary:**

Combretastatin A4-phosphate is a chemotherapeutic drug which has been evaluated for treatment of solid canine tumors. Previous studies reported cardiotoxic effects based on changes in cardiac troponin I measurements, blood pressure, and electrocardiography. We evaluated the cardiotoxic effect by two-dimensional speckle tracking echocardiography. This advanced imaging technique analyzes global and regional myocardial function and is used as the gold-standard for the assessment of cardiac function in human patients receiving chemotherapy. We found that certain strain measurements were significantly decreased 24 h after the administration of combretastatin A4-phosphate and that these changes were correlated with an increase in cardiac troponin I. Our results suggest that two-dimensional speckle tracking may be useful for the early detection of cardiac dysfunction in canine cancer patients as well as promising during follow-up.

**Abstract:**

Combretastatin A4-phosphate (CA4P) is a vascular disrupting agent that was recently described for the treatment of solid canine tumors. Conventional echocardiography and pulsed wave tissue Doppler imaging did not reveal cardiotoxicity in dogs, however, the gold standard for assessing myocardial damage in humans receiving cardiotoxic chemotherapeutics is two-dimensional speckle-tracking echocardiography. The current study evaluated the cardiotoxic effect of a single dose of CA4P in dogs using peak systolic strain measurements and the variability of these measurements. Echocardiographic examinations of seven healthy beagles and five canine cancer patients that received CA4P were retrospectively reviewed. Peak systolic regional longitudinal strain (LSt), peak systolic regional circumferential strain (CSt), and peak systolic regional radial strain (RSt) were measured before and 24 h after administration of CA4P. Peak systolic strain measurements were compared to serum cardiac troponin I (cTnI). To quantify intra- and inter-observer measurement variability, seven echocardiographic examinations were selected and each strain parameter was measured by three observers on three consecutive days. After CA4P administration, the median LSt and CSt values decreased by 21.8% (*p* = 0.0005) and 12.3% (*p* = 0.002), respectively, whereas the median RSt values were not significantly different (*p* = 0.70). The decrease in LSt was correlated with increased serum cTnI values (Spearman rho = −0.64, *p* = 0.02). The intra-observer coefficients of variation (CV) were 9%, 4%, and 13% for LSt, CSt, and RSt, respectively, while the corresponding interobserver CVs were 11%, 12%, and 20%. Our results suggest that regional peak systolic strain measurements may be useful for the early detection of cardiotoxicity that is caused by vascular disrupting agents and that LSt may be promising for the follow-up of canine cancer patients.

## 1. Introduction

Combretastatin A4-phosphate (CA4P), also called fosbretabulin, is a chemotherapeutic drug that has recently been evaluated in healthy dogs and canine cancer patients [1,2,3]. This compound belongs to the class of vascular disrupting agents which induce selective destruction of immature blood vessels that typically supply tumors [1]. Immature vessels are characterized by constant remodeling, increased permeability, and the absence of peri-endothelial cell recruitment and innervation [4,5]. Microtubules play a predominant role in maintaining the shape and attachment of the newly formed endothelial cells in the immature vessels [6]. Following intracellular uptake and dephosphorylation of CA4P to CA4, the latter targets these microtubules by binding reversibly to tubulin at the colchicine-binding site, leading to tubulin depolymerization [7,8]. As a result, microtubule assembly in tumor neovasculature is inhibited, causing distortion and detachment of the immature proliferating endothelial cells [4,8]. Rapid collapse and regression of tumor vessels prevent further inflow of factors (nutrients, growth factors, metabolites, inflammatory mediators, and oxygen) that are indispensable for the survival and division of tumor cells, leading to necrosis of the neoplastic cells [4,5,8].

CA4P is administered intravenously and has a relatively short half-life of ca. 10–27 min. Therefore, a one-time administration of CA4P is likely to induce minimal side effects, which are considered reversible [9,10,11]. However, dose-dependent non-cardiovascular and cardiovascular adverse effects have been described in human and veterinary studies [1,2,3,12,13,14]. Cardiotoxicity can occur secondary to disturbance of the myocardial microcirculation or as a direct toxic effect on cardiomyocytes [15,16]. Cardiovascular adverse effects include a transient increase in cardiac troponin I (cTnI) levels, transient systemic arterial hypertension, and electrocardiographic changes such as sinus tachycardia, sinus bradycardia, and ventricular arrhythmias [1,2,3,12,13,14,17,18]. Additionally, myocardial ischemia and rare cases of myocardial stunning have been reported in people after CA4P administration [13,18,19].

Cancer therapy-related cardiac dysfunction (CTRCD) has recently been recognized as a leading cause of morbidity and mortality in cancer survivors in human medicine [20,21,22]. For comprehensive analysis of the myocardial function, two-dimensional speckle-tracking echocardiography (2D-STE) is the most widely used technique in human medicine and is used to detect early myocardial damage in patients receiving potentially cardiotoxic chemotherapy [20,21,22,23]. Left ventricular strain measurements study the myocardial deformation in the longitudinal, circumferential, and radial dimensions, which are very sensitive for the detection of myocardial dysfunction, even before the left ventricular ejection fraction declines [20,21,22,23,24,25,26,27,28]. The early detection of cardiac dysfunction is warranted since further myocardial damage can be avoided by changing the chemotherapy protocol and/or starting cardiac medication. This results in fewer cardiac complications, reduced medical costs, and improved quality of life for cancer patients and is, therefore, an important factor in the follow-up protocol [20,21,29,30].

Cardiotoxicity after CA4P administration in dogs has been evaluated using conventional echocardiographic measurements. These include fractional shortening and ventricular diameters measured from M-mode images and ventricular volumes and ejection fraction measured by the Simpson method of discs on two-dimensional images to assess left ventricular systolic function. Additionally, pulsed wave tissue Doppler imaging of the left ventricular free wall has been investigated. No significant differences between pre- and post-CA4P administration were detected [2,3]. However, these conventional echocardiographic measurements are not sensitive in detecting early subclinical ventricular dysfunction [20,23]. Until now, 2D-STE measurements, as used in human medicine, have not been implemented in dogs to evaluate CTRCD.

The aim of this study was to determine cardiotoxicity after administration of a single intravenous dose of CA4P in dogs by evaluating peak systolic left ventricular strain measurements. The second aim of the study was to evaluate the intra- and inter-observer variability of such strain measurements.

## 2. Materials and Methods

### 2.1. Case Selection

The medical records from dogs that were included in the two previously published studies by Abma et al. [2,3] were retrospectively reviewed. These dogs received one dose of intravenous CA4P and underwent a complete transthoracic echocardiographic examination before inclusion and 24 h after administration of CA4P. During image acquisition, the dogs were gently restrained first in right and then in left lateral recumbency and did not require sedation. The dogs were breathing normal during the examination. Echocardiographic images were obtained by one investigator (P.S., board-certified cardiologist), using a Vivid 7 (GE Medical Systems, Chicago, IL, USA) and 1.5–3.6 MHz and 3.5–8 MHz phased-array transducers with continuous ECG recording. Conventional echocardiography and pulsed wave tissue Doppler imaging (measured by a board-certified cardiologist, P.S.) were within the reference interval on both examinations (before and 24 h after CA4P administration) for each dog, as previously published. Cases were excluded from the current study when the image quality was poor, the frame rate was below 60 frames per second, or the right parasternal short axis view at the level of the papillary muscles and left apical four-chamber views were not both available for each exam. Of those dogs that were retained, age, breed, sex, and body weight were collected. In addition, serum cTnI levels (I-Stat, Abbott Axsym System, Abbott AG, Baar, Switzerland), non-invasive Doppler measurements of the systolic arterial blood pressure (according to the ACVIM consensus guidelines [31]), and six-lead five-minute electrocardiography (before and after CA4P administration) were collected and when available, also the results of a 24-h-Holter monitoring after CA4P administration.

### 2.2. Echocardiographic Measurements

**Figure 1:**
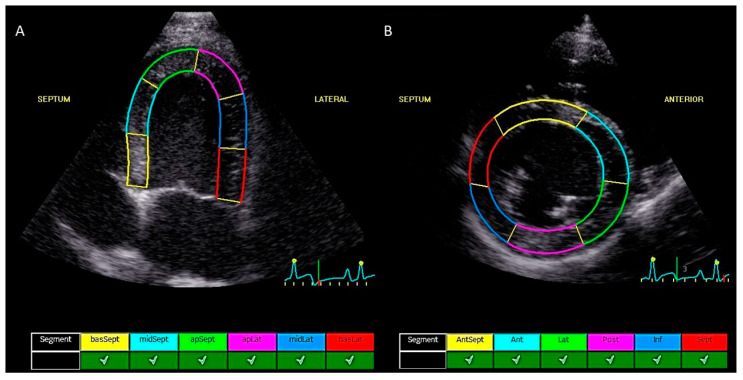
Representative two-dimensional speckle tracking echocardiographic images that were obtained from a healthy Beagle of 7.5-year old before administration of combretastatin A4-phosphate. (**A**) Regional peak systolic longitudinal strain tracking on a left apical four-chamber view at the end of systole dividing the left ventricular myocardium into six equal segments: basal septal (yellow), mid septal (light blue), apical septal (green), apical lateral (purple), mid-lateral (dark blue), and basal lateral (red). (**B**) Regional peak systolic circumferential strain and radial strain tracking on a right parasternal short axis view at the end of diastole at the level of the papillary muscles dividing the left ventricular myocardium in six equal segments: anteroseptal (yellow), anterior (light blue), lateral (green), posterior (purple), inferior (dark blue), and septal (red). Below both echocardiographic images (**A**,**B**) all myocardial segments are recognized as having adequate tracking quality and can be approved for the calculation of strain values.

**Figure 2:**
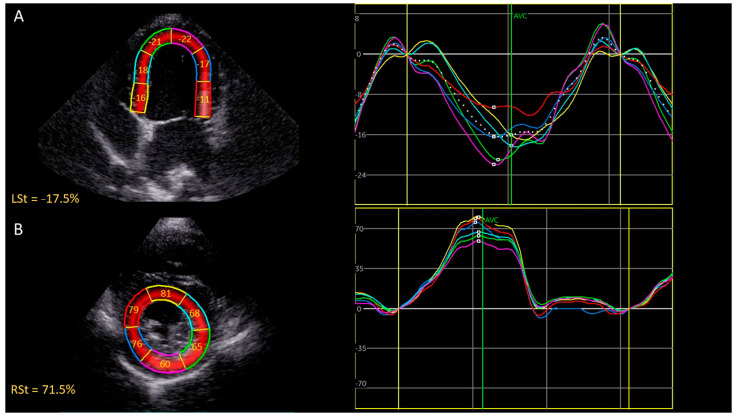
Representative two-dimensional speckle tracking echocardiography of the left ventricle in a 7-year-old healthy Beagle 24 h after CA4P administration. The images on the left represent the tracking of the myocardial wall in the long axis (**A**) and short axis (**B**). The images on the right side represent all six segmental strain curves (colored lines) from each myocardial region and one regional strain curve (average of segmental strains, dotted line). (**A**) Regional left ventricular longitudinal strain curves during one cardiac cycle. The peak systolic longitudinal strain is measured during the ejection phase. (**B**) Regional left ventricular radial strain curves during one cardiac cycle. The peak systolic radial strain is measured during the ejection phase. Abbreviations: AVC, aortic valve closure; LSt, regional peak systolic longitudinal strain; RSt, regional peak systolic radial strain.

The echocardiographic data were analyzed offline using the commercial software program EchoPac (GE Medical Systems, version 203). Measurements were performed by a single investigator (G.M.). The investigator was blinded to the patient’s identity and the time of recording (before versus after CA4P administration). All echocardiographic examinations were selected in a random order by a randomization program. The endocardial border of the left ventricle was manually defined by selecting points on the endocardium, followed by an automatic frame-by-frame tracking of speckle patterns at end systole. The software automatically divided the myocardium into 6 segments in accordance with standard segmentation models used in humans, as previously described [28,32,33]. Manual adjustment of the region of interest was performed to select the myocardium. When segments were marked by the software as being of inadequate tracking quality, the procedure was repeated on a similar image. Only cardiac cycles with five or more segments of adequate tracking quality and without arrhythmias were included. From the left apical four-chamber view, the six segmental values were averaged to obtain peak systolic regional longitudinal strain (LSt) (Figure 1A). Similarly, the six segmental values from the right parasternal short axis view (at the level of the papillary muscles) were averaged to obtain peak systolic regional circumferential strain (CSt) and peak systolic regional radial strain (RSt) (Figure 1B). The measurements were repeated in three consecutive cardiac cycles. These three values were averaged to obtain the final peak systolic strain value for each strain parameter before and after CA4P administration. Peak systolic longitudinal strain was measured only on the left apical four-chamber view (septo-lateral left ventricular walls) and CSt and RSt were only measured on the right parasternal short axis view at the level of the papillary muscles (mid-left ventricle). Images were not available to measure three different image planes for each strain parameter, so a bull’s eye could not be obtained. The peak systolic strain was measured, which was defined as the maximal deflections of the strain curves during the ejection phase, the phase between the end of the QRS complex, and the end of the T-wave derived from the simultaneously recorded ECG (previously marked, based on spectral Doppler images of aortic velocity and mitral inflow profile to determine aortic and mitral valve opening and closure) (Figure 2). Manual adjustments in the results section were made when the automatic detection of peak systolic phase was incorrect. The peak systolic deflection of the strain curves describe the magnitude of myocardial deformation during systole relative to the initial length or thickness at the end of diastole, expressed as a percentage [34,35]. Therefore, the LSt and CSt are displayed as negative values and the RSt is displayed as a positive value. For the remainder of this article, absolute values of the strain values will be used without units. The relative change in the strain values after CA4P administration will be expressed as a percentage. The values used for statistical analysis were the average value from three consecutive strain measurements on three different cardiac cycles.

### 2.3. Intra- and Inter-Observer Measurement Variability

The intra- and inter-observer measurement variability with the coefficient of variation (CV) for the three different peak systolic strain parameters was evaluated based on seven echocardiographic examinations. On these examinations, all three peak systolic strain parameters (LSt, CSt, and RSt) were measured by three different operators (P.S., G.M., and A.H.). The investigators were unaware of the results of previous measurements. All three operators repeated the peak systolic strain measurements individually on three consecutive days.

### 2.4. Statistical Analysis

The normality of the data was determined using QQ plots. The presence of significant differences between measurements before and after CA4P administration was assessed using a Wilcoxon sign rank test for paired measurements. The correlation between the relative change in LSt, CSt, and RSt and the absolute change in the serum cTnI level was assessed with a Spearman Rho test. To quantify the intra- and inter-observer measurement variation, a random effect model with dog and observer nested in dog as random effects and peak systolic strain parameters as outcome was fitted for LSt, CSt, and RSt. The CV for the intra- and inter-observer measurement variability was determined by taking the residual variance, and the residual variance together with the added variance by observer, respectively, and dividing these by the mean of the measurements. All analyses were performed via R version 4.2.0. Significance was set at α ≤ 0.05.

## 3. Results

### 3.1. Population Characteristics

Echocardiographic images of 10 healthy Beagles and nine canine cancer patients were reviewed. Inadequate image quality (n = 2), missing pre- or post-administration echocardiographic examinations (n = 3), and unavailability of image planes that were needed for peak systolic strain measurements (n = 2) led to the exclusion of three of the ten healthy beagles and four of the nine cancer patients. The data of the remaining seven healthy beagles and five cancer patients were included for final data analysis. In two of the beagles that were included, the right parasternal short axis view was not of sufficient quality for one of the two timepoints, so CSt and RSt were not measured for these dogs. Signalment, dose of CA4P administered, peak systolic strain values, and the serum cTnI levels are summarized in Table 1. A total of five dog breeds were included. Of the dogs, eight were male and three dogs were female. The dogs had a median age of 7.5-years (range 4–14 years) and a median body weight of 15.8 kg (range 7.3–35.0 kg). The dose of CA4P that was administered varied: 50 mg/m^2^ in two dogs, 75 mg/m^2^ in eight dogs, and 100 mg/m^2^ in two dogs. All cancer patients that were included were diagnosed with a solid tumor. Tumor type was defined by analysis of at least two 4–6 mm punch biopsies. A total of four of the included patients were diagnosed with a tumor of mesenchymal origin of which three had a soft tissue sarcoma and one had a chondrosarcoma. One case was diagnosed with a round cell tumor (mastocytoma).

### 3.2. Two-Dimensional Speckle Tracking Echocardiographic Measurements

**Figure 3:**
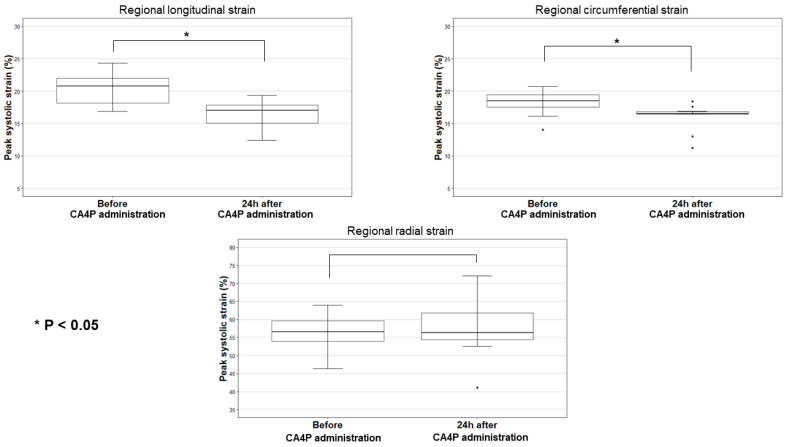
Boxplots presenting the regional peak systolic longitudinal strain, regional peak systolic circumferential strain, and regional peak systolic radial strain of all dogs before and 24 h after combretastatin A4-phosphate (CA4P) administration. Regional peak systolic longitudinal strain and regional peak systolic circumferential strain are significantly different when comparing the measurements before vs. 24 h after CA4P administration (*p* < 0.05). Abbreviations: CA4P, combretastatin A4-phosphate.

The left ventricular regional peak systolic strain for each dog before and 24 h after CA4P administration is listed in Table 1. The median values of LSt, CSt, and RSt before CA4P administration were 20.8 (range 16.9–24.3), 18.5 (range 14.0–20.7), and 56.5 (range 46.4–64.0), respectively (Table 2). Boxplots of the peak systolic strain measurements are shown in Figure 3. The median values of LSt decreased by 4.5 (range 0.7–6.2, *p* < 0.001) 24 h after CA4P administration, corresponding to a 21.8% decrease in absolute value. In addition, a decrease in the median CSt values by 2.3 (range 0.3–4.3) after CA4P administration was found (*p* = 0.002), corresponding to 12.3% decrease in the absolute CSt value. The median value of RSt increased by 1.6 (range −9.1–11.4), but this change was not statistically significant (*p* = 0.70) (Table 2). The LSt and CSt values decreased in every individual dog, whereas RSt increased in some, but decreased in others. Both dogs that received 100 mg/m^2^ and three dogs that received 75 mg/m^2^ had LSt values that decreased by more than 4.5 after CA4P administration (Table 1).

**Figure 4:**
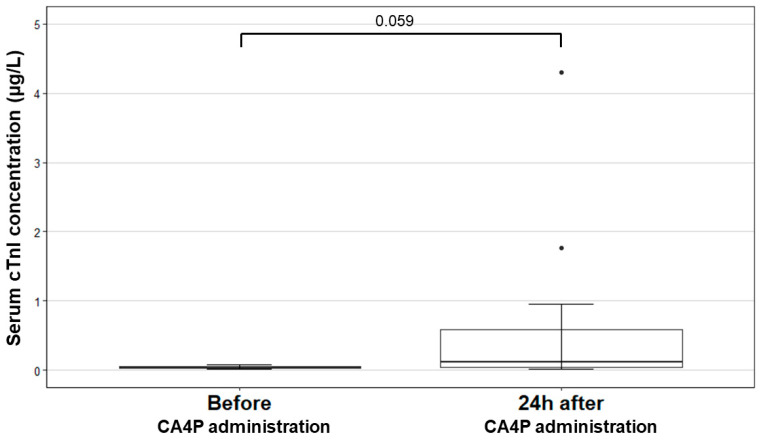
Boxplots presenting the serum cardiac troponin I (cTnI) of all dogs before and 24 h after combretastatin A4-phosphate (CA4P) administration (reference interval 0.01–0.11 µg/L). cTnI was not significantly different (*p* = 0.059) between the two time-points for the complete study group. Abbreviations: CA4P, combretastatin A4-phosphate; cTnI, cardiac troponin I.

Serum cTnI values are presented in Table 1. The serum cTnI at baseline was within the reference interval (0.01–0.11 µg/L) [36] for all dogs that were included. The median serum cTnI values increased from 0.04 µg/L (range 0.02–0.08 µg/L) before administration to 0.12 µg/L (range 0.01–4.3 µg/L) 24 h after CA4P administration. However, this mean value is above the reference interval and this change was not statistically significant (*p* = 0.059). The serum cTnI levels were within the reference interval (0.01–0.11 µg/L) in the two dogs that received 50 mg/m^2^ but were elevated in four of the eight dogs that received 75 mg/m^2^ and in the two dogs that received 100 mg/m^2^. A boxplot of the differences in the serum cTnI levels for the dogs’ pre-treatment vs. 24 h post-treatment is presented in Figure 4. A significant, negative correlation was found between the relative changes in LSt—and the absolute change in serum cTnI (Spearman Rho = −0.64, *p* = 0.02). There was no significant correlation between the relative changes in CSt and the absolute change in serum cTnI (*p* = 0.38), nor between the changes in RSt and serum cTnI (*p* = 0.95) (Table 3).

### 3.3. Inter- and Intra-Observer Measurement Variability

The intra-observer (between-day) measurement variability, expressed as the CV, was 9%, 4%, and 13% for LSt, CSt, and RSt, respectively. Similarly, the inter-observer measurement variability was 11%, 12%, and 20% for LSt, CSt, and RSt, respectively.

### 3.4. Blood Pressure and ECG Data

Of the 12 dogs that were included in this study, none had systemic arterial hypertension (systolic arterial blood pressure > 160 mmHg) [31] before CA4P administration. The blood pressure was normal (systolic arterial blood pressure < 140 mmHg) [31] in nine dogs and three dogs had a systolic arterial blood pressure of 150 mmHg before CA4P administration. After CA4P administration, no abnormalities in blood pressure were detected in any of the dogs with serum cTnI values within the reference interval. Transient systemic arterial hypertension (systolic arterial blood pressure > 160 mmHg) [31] was observed in the two beagles that received 100 mg/m^2^ of CA4P and in one of the three canine patients with increased cTnI values after receiving 75 mg/m^2^ of CA4P. Ventricular arrhythmias were seen in the three beagles with increased serum cTnI values (two that were treated with 100 mg/m^2^ and one that was treated with 75 mg/m^2^ of CA4P). Transient bradycardia was present in three canine patients with increased serum cTnI values after receiving 75 mg/m^2^ of CA4P. Amongst the dogs with serum cTnI values within the reference interval after CA4P administration, one patient developed transient bradycardia and one developed transient tachycardia after CA4P administration. Both received 75 mg/m^2^ of CA4P.

## 4. Discussion

Our study is the first to evaluate the use of peak systolic strain measurements for the detection of CTRCD in dogs that were treated with vascular disrupting agents, which are potentially cardiotoxic. The results show a significant decrease in LSt and CSt after the administration of a single dose of CA4P in dogs. The LSt values decreased with an average of 21.8% relative to the values before administration. Furthermore, a significant negative correlation was demonstrated between LSt and serum cTnI after administration of CA4P.

Possible cardiotoxicity due to CA4P administration has been described in veterinary medicine as increased serum cTnI levels, transient systemic arterial hypertension, sinus bradycardia, sinus tachycardia, and ventricular arrhythmias [2,3]. Nevertheless, conventional echocardiography and pulsed wave tissue Doppler imaging failed to detect CTRCD after a single dose of CA4P in dogs [2,3,37,38]. The advantage of 2D-STE is that myocardial dysfunction can be detected in earlier stages compared to conventional echocardiography [39,40]. Also, 2D-STE is considered to be angle-independent, which allows evaluation along different spatial orientations. Additionally, 2D-STE is independent of passive, in-plane translational movement and tethering of myocardial segments [4], which permits the differentiation between active myocardial deformation and passive myocardial movement [41,42]. In human medicine, the cardio-oncology expert consensus of the American Society of Echocardiography and European Association for Cardiovascular Imaging recommends routine use of 2D-STE, in particular global longitudinal strain (GLS), as the gold standard for monitoring patients during cancer therapy to assess early development of CTRCD [20,21,22,29,30,43]. The preference for GLS is not surprising as longitudinal myocardial fibers are particularly susceptible to ischemia because the myocardium is perfused from the epicardium to the endocardium [44,45]. An early reduction of GLS may forecast the development of myocardial dysfunction and subsequent cardiotoxicity [20,22,29,30,43] and may be a predictor of cardiovascular morbidity and mortality [21]. A relative reduction of >15% in GLS from the baseline is considered a significant change to detect cardiotoxicity in human patients [21,43,46]. Relative changes in GLS < 8% are regarded as inconclusive [21]. Our results showed an average decrease of approximately 22% in LSt relative to the initial value before CA4P administration and this decrease was significantly negatively correlated with the serum cTnI values. Increased serum cTnI values can detect early myocardial injury and are prognostically negative in human and veterinary cancer patients that are receiving chemotherapy [47,48,49]. This might suggest that a decrease in LSt may be useful to detect CTRCD and may thus also have a prognostic value in dogs that are receiving chemotherapy, as is described in human medicine [21,22]. Further prospective studies are required to assess the clinical utility of LSt and GLS for therapeutic monitoring of canine cancer patients, ideally to define similar prognostic cut-off values as described in human medicine. Global circumferential strain (GCS) and global regional strain (GRS) are reported to be less reproducible than GLS in people and currently have limited clinical application in human cardio-oncology [50]. Although a relative change in CSt was observed in the dogs after CA4P administration compared to before, it was much smaller than for LSt and it was not correlated with changes in serum cTnI. These findings suggest that also in dogs CSt may be less useful for early detection of CTRCD.

We observed significant changes in LSt after CA4P administration in the majority of our dogs. In human medicine, cardiac dysfunction is only reported in patients who received dose levels of ≥50 mg/m^2^ CA4P [17,37]. All the dogs in the current study received ≥50 mg/m^2^. Lower dosages may induce more subtle changes in strain values.

The reported reference intervals for 2D-STE measurements in healthy dogs are relatively wide, with mean and median values ranging from 14.8% to 26.3% for GLS, 15.4% to 20.9% for GCS, and 31.9% to 52.4% for GRS, respectively [23,32,33,51,52]. These studies assessed reference intervals in relatively small populations (20–100 dogs) and various breeds. The median values of 2D-STE measurements in this study match those in previous reports, with a similarly wide range. On the contrary, in human medicine the reference intervals for 2D-STE measurements are relatively narrow [53,54]. The reference interval for GLS values in humans is reported to be >18%. GLS values between 16% and 18% are borderline, whereas values < 16% are abnormally low [55]. Other disadvantages of 2D-STE are their dependence on the age and weight and on the vendor and software that is used for analysis [51,56,57,58]. In dogs specifically, 2D-STE is also dependent on the breed. Additionally, 2D-STE is not available in all echocardiography devices, offline analysis requires high-quality image data with a high frame rate and measuring is time consuming compared to conventional echocardiography. Besides, strain measurements are dependent of cardiac loading conditions. So, they can estimate the systolic function but do not directly measure contractility [26,35]. As an alternative in patients with poor sonographic window or in poor quality images, mitral annular plane systolic excursion (MAPSE) may be considered as a marker to detect early left ventricular systolic dysfunction. This measurement has a good correlation with GLS and has an increased sensitivity over traditional methods for measuring systolic function [59,60].

Besides the relatively wide reference interval, previous veterinary studies showed variable results of reproducibility of 2D-STE measurements. Intra- (between-day and within-day) and inter-observer measurement variability for GLS and GCS were both < 15% in various studies [23,33,52,61,62]. The intra- and inter-observer measurement variability of GRS values have been described to show generally a greater measurement variability compared to GLS and GCS [23,63]. However, the studies of Chetboul et al. [31] and Suzuki et al. [52] reported less intra-observer measurement variability of the GRS measurements compared to other strain measurements. Strain values with CV < 15% are considered clinically acceptable [64,65]. According to this cut-off, the intra-observer variability of all strain measurements in our study was clinically acceptable. The inter-observer variability was clinically acceptable for LSt and CSt but not for RSt. These values of reproducibility are in accordance with the previous findings in veterinary medicine [23,33,51,52,56,61].

The limitations of this study are mostly related to the retrospective nature. First of all, echocardiographic evaluation was only available 24 h after administration of CA4P without follow-up examinations. Longer echocardiographic follow-up would have been interesting to see if strain parameters changed over time. As the serum cTnI levels of the dogs normalized within a few weeks after administration [2,3], it is possible that the strain measurements would have normalized as well. Second, to the authors knowledge, this is the first study looking at changes in the 2D-STE values after administration of a vascular disrupting agent. The sample size of this study was rather small. Nevertheless, significant changes in 2D-STE values due to CA4P administration were detected, suggesting the presence of CTRCD. Further studies are necessary to confirm the usefulness of this diagnostic tool in larger study samples. Third, the dogs in this study received different single doses of CA4P and no conclusions can be made about the relationship between the dose and cardiotoxicity or about the effect of cumulative dosing. Fourth, we did not have the images available to measure the GLS, GCS, and GRS averaged from three different image planes. Consequently, no bull’s eye profile could be assessed, so no information about the exact position of myocardial damage was available. However, in dogs, only one plane is generally used to define the longitudinal strain and, therefore, the left parasternal apical location is typically chosen [34,61]. In human medicine, bull’s eye is commonly assessed in the follow-up of oncology patients. Further studies investigating the different views are warranted [41,66,67]. Lastly, we did not evaluate the reproducibility of the complete 2D-STE technique itself because we performed repeated measurements on the same, previously recorded images. Therefore, we assessed the variability of the measurements. To assess the variability of the technique itself it would be necessary to record separate images from the same dog in a prospective design to perform the strain measurements.

## 5. Conclusions

In conclusion, this study shows that LSt detects early cardiotoxic effects after the administration of a single high dose of CA4P. These measurements show an acceptable intra- and interobserver measurement variability. LSt may be useful for the early detection of CTRCD after administration of vascular disrupting agents. Further prospective studies are warranted to confirm the current results and to assess the potential application of advanced echocardiographic tools in the monitoring of veterinary cancer patients.

## Figures and Tables

**Table 1:** This table represents the demographic data of the dogs that were included in the study. Signalment of dogs that were treated with combretastatin A4-phosphate (CA4P) and the dose that was administered to each dog are presented on the left side of this table. The right side presents the results of left ventricular regional peak systolic strain values before (pre) and 24 h after (post) CA4P administration as well as the results of serum cardiac troponin I before (pre) and 24 h after (post) CA4P administration. Abbreviations: Am. Staff., American Staffordshire Terrier; CA4P, combretastatin A4-phosphate; CSt, regional peak systolic circumferential strain; cTnI, cardiac troponin I; F, Female; Fn, Female neutered; LSt, regional peak systolic longitudinal strain; Mn, male neutered; RSt, regional peak systolic radial strain; 2D-STE, two-dimensional speckle tracking echocardiography.

Dog	Breed	Age (Years)	Sex	Weight (kg)	Dose CA4P (mg/m^2^)	2D-STE Parameters						cTnI (µg/L)	
						LSt		CSt		RSt		Pre	Post
						Pre	Post	Pre	Post	Pre	Post		
1	Beagle	7.5	Mn	17.0	50	22.8	17.8	20.7	16.4	54.1	59.8	0.04	0.01
2	Beagle	7.5	Mn	13.0	50	21.3	18.2	20.4	16.7	55.8	54.7	0.05	0.04
3	Beagle	8	Fn	8.0	75	17.6	14.2	17.1	16.5	57.3	55.0	0.02	0.03
4	Beagle	8.5	Fn	7.3	100	24.3	19.4	18.1	/	55.7	/	0.03	0.40
5	Beagle	7	Mn	17.8	100	22.9	17.8	20.1	16.9	64.0	72.1	0.02	4.30
6	Beagle	7.5	Mn	19.8	75	21.7	17.2	18.7	18.4	60.0	54.2	0.02	0.16
7	Beagle	7.5	Mn	14.5	75	21.3	18.0	19.2	/	63.5	/	0.03	0.02
8	Am. Staff.	14	Mn	14.0	75	16.9	15.4	16.1	13.0	53.5	62.5	0.08	0.08
9	Golden Retriever	10	Mn	35.0	75	18.4	12.4	18.8	17.6	46.4	57.7	0.05	1.77
10	Münsterländer	4	Fn	22.0	75	17.5	16.8	17.7	16.5	58.3	62.5	0.04	0.04
11	Am. Staff.	10	Mn	35.0	75	20.3	14.1	18.2	16.5	59.5	52.5	0.05	0.95
12	Whippet	7	Fn	13.1	75	19.9	15.4	14.0	11.2	50.2	41.1	0.08	0.46

**Table 2:** Changes in the left ventricular regional peak systolic strain values from before CA4P administration in comparison with 24 h after CA4P administration. *p*-values were calculated using the paired Wilcoxon test. Abbreviations: CA4P, combretastatin A4-phosphate; CSt, regional peak systolic circumferential strain; LSt, regional peak systolic longitudinal strain; RSt, regional peak systolic radial strain; 2D-STE, two-dimensional speckle tracking echocardiography.

2D-STE Parameter	Median Value before CA4P Administration	Range	Median Absolute Change 24 h after CA4P Administration	Range	*p*-Value
LSt	20.8	16.9–24.0	4.5	0.7–6.2	<0.001
CSt	18.5	14.0–20.7	2.3	0.3–4.3	0.002
RSt	56.7	46.4–64.0	1.6	−9.1–11.4	0.70

**Table 3:** Spearman correlation of the two-dimensional speckle tracking echocardiography (2D-STE) parameters regional peak systolic longitudinal strain (LSt), regional peak systolic circumferential strain (CSt), and regional peak systolic radial strain (RSt) with serum cardiac troponin I (cTnI) levels. Abbreviations: cTnI, cardiac troponin I; CSt, regional peak systolic circumferential strain; LSt, regional peak systolic longitudinal strain; RSt, regional peak systolic radial strain; 2D-STE, two-dimensional speckle tracking echocardiography.

2D-STE Parameter	Spearman Rho Value for Correlation with cTnI	*p*-Value
LSt	−0.64	0.02
CSt	0.31	0.38
RSt	−0.02	0.95

## Data Availability

The data that are presented in this study are available on request from the corresponding author. An abstract of the preliminary data was presented on the 24th of June 2022 at the American College of Veterinary Internal Medicine congress, Texas, USA.

## Abbreviations

CA4P	Combretastatin A4-phosphate
CTRCD	Cancer therapy-related cardiac dysfunction
CV	Coefficient of variation
2D-STE	Two-dimensional speckle tracking echocardiography
cTnI	Cardiac troponin I
GCS	Global circumferential strain
GLS	Global longitudinal strain
GRS	Global radial strain
LSt	Peak systolic regional longitudinal strain
CSt	Peak systolic regional circumferential strain
RSt	Peak systolic regional radial strain

## References

[1] Abma E., Daminet S., Smets P., Ni Y., de Rooster H. (2015). Combretastatin A4-phosphate and its potential in veterinary oncology: A review. Vet. Comp. Oncol..

[2] Abma E., De Spiegelaere W., Vanderperren K., Stock E., Van Brantegem L., Cornelis I., Daminet S., Ni Y., Vynck M., Verstraete G. (2018). A single dose of intravenous combretastatin A4-phosphate is reasonably well tolerated and significantly reduces tumour vascularization in canine spontaneous cancers. Vet. Comp. Oncol..

[3] Abma E., Smets P., Daminet S., Cornelis I., De Clercq K., Ni Y., Vlerick L., de Rooster H. (2018). A dose-escalation study of combretastatin A4-phosphate in healthy dogs. Vet. Comp. Oncol..

[4] Griggs J., Metcalfe J.C., Hesketh R. (2001). Targeting tumour vasculature: The development of combretastatin A4. Lancet Oncol..

[5] Vincent L., Kermani P., Young L.M., Cheng J., Zhang F., Shido K., Lam G., Bompais-Vincent H., Zhu Z., Hicklin D.J. (2005). Combretastatin A4 phosphate induces rapid regression of tumor neovessels and growth through interference with vascular endothelial-cadherin signaling. J. Clin. Investig..

[6] Bergers G., Benjamin L.E. (2003). Tumorigenesis and the angiogenic switch. Nat. Rev. Cancer.

[7] Pettit G.R., Singh S.B., Hamel E., Lin C.M., Alberts D.S., Garcia-Kendall D. (1989). Isolation and structure of the stong cell growth and tubulin inhibitor combretastatin A-4. Experientia.

[8] Liu L., O’kelly D., Schuetze R., Carlson G., Zhou H., Trawick M.L., Pinney K.G., Mason R.P. (2021). Non-invasive evaluation of acute effects of tubulin binding agents: A review of imaging vascular disruption in tumors. Molecules.

[9] Hamel E. (1996). Antimitotic natural products and their interactions with tubulin. Med. Res. Rev..

[10] Tozer G.M., Kanthou C., Baguley B.C. (2005). Disrupting tumour blood vessels. Nat. Rev. Cancer.

[11] Ibrahim M.A., Do D.V., Sepah Y.J., Shah S.M., Van Anden E., Hafiz G., Donahue J.K., Rivers R., Balkissoon J., Handa J.T. (2013). Vascular disrupting agent for neovascular age related macular degeneration: A pilot study of the safety and efficacy of intravenous combretastatin a-4 phosphate. BMC Pharmacol. Toxicol..

[12] Stevenson J.P., Rosen M., Sun W., Gallagher M., Haller D.G., Vaughn D., Giantonio B., Zimmer R., Petros W.P., Stratford M. (2003). Phase I trial of the antivascular agent combretastatin A4 phosphate on a 5-day scedule to patients with cancer: Magnetic resonance imaging evidence for altered tumor blood flow. J. Clin. Oncol..

[13] Dowlati A., Robertson K., Cooney M., Petros W.P., Stratford M., Jesberger J., Rafie N., Overmoyer B., Makkar V., Stambler B. (2002). A phase I pharmacokinetic and translational study of the novel vascular targeting agent combretastatin A-4 phosphate on a single-dose intravenous schedule in patients with advanced cancer. Cancer Res..

[14] Rustin G.J.S., Galbraith S.M., Anderson H., Stratford M., Folkes L.K., Sena L., Gumbrell L., Price P.M. (2003). Phase I clinical trial of weekly combretastatin A4 phosphate: Clinical and pharmacokinetic results. J. Clin. Oncol..

[15] Tochinai R., Nagata Y., Ando M., Hata C., Suzuki T., Asakawa N., Yoshizawa K., Uchida K., Kado S., Kobayashi T. (2016). Combretastatin A4 disodium phosphate-induced myocardial injury. J. Toxicol. Pathol..

[16] Tochinai R., Komatsu K., Murakami J., Nagata Y., Ando M., Hata C., Suzuki T., Kado S., Kobayashi T., Kuwahara M. (2018). Histopathological and functional changes in a single-dose model of combretastatin A4 disodium phosphate-induced myocardial damage in rats. J. Toxicol. Pathol..

[17] He X., Li S., Huang H., Li Z., Chen L., Ye S., Huang J., Zhan J., Lin T. (2011). A pharmacokinetic and safety study of single dose intravenous combretastatin A4 phosphate in Chinese patients with refractory solid tumours. Br. J. Clin. Pharmacol..

[18] Subbiah I.M., Lenihan D.J., Tsimberidou A.M. (2011). Cardiovascular Toxicity Profiles of Vascular-Disrupting Agents. Oncologist.

[19] Bhakta S., Flick S.M., Cooney M.M., Greskovich J.F., Gilkeson R.C., Remick S.C., Ortiz J. (2009). Myocardial stunning following combined modality combretastatin-based chemotherapy: Two case reports and review of the literature. Clin. Cardiol..

[20] Oikonomou E.K., Kokkinidis D.G., Kampaktsis P.N., Amir E.A., Marwick T.H., Gupta D., Thavendiranathan P. (2019). Assessment of Prognostic Value of Left Ventricular Global Longitudinal Strain for Early Prediction of Chemotherapy-Induced Cardiotoxicity: A Systematic Review and Meta-analysis. JAMA Cardiol..

[21] Plana J.C., Galderisi M., Barac A., Ewer M.S., Ky B., Scherrer-Crosbie M., Ganame J., Sebag I.A., Agler D.A., Badano L.P. (2014). Expert consensus for multimodality imaging evaluation of adult patients during and after cancer therapy: A report from the American society of echocardiography and the European association of cardiovascular imaging. J. Am. Soc. Echocardiogr..

[22] Lyon A.R., López-Fernández T., Couch L.S., Asteggiano R., Aznar M.C., Bergler-Klein J., Boriani G., Cardinale D., Cordoba R., Cosyns B. (2022). 2022 ESC Guidelines on cardio-oncology developed in collaboration with the European Hematology Association (EHA), the European Society for Therapeutic Radiology and Oncology (ESTRO) and the International Cardio-Oncology Society (IC-OS). Eur. Heart J..

[23] Hamabe L., Mandour A.S., Shimada K., Uemura A., Yilmaz Z., Nagaoka K., Tanaka R. (2021). Role of two-dimensional speckle-tracking echocardiography in early detection of left ventricular dysfunction in dogs. Animals.

[24] Blessberger H., Binder T. (2010). Two dimensional speckle tracking echocardiography: Basic principles. Heart.

[25] Bijnens B.H., Cikes M., Claus P., Sutherland G.R. (2009). Velocity and deformation imaging for the assessment of myocardial dysfunction. Eur. J. Echocardiogr..

[26] Dandel M., Hetzer R. (2009). Echocardiographic strain and strain rate imaging—Clinical applications. Int. J. Cardiol..

[27] Biswas M., Sudhakar S., Nanda N.C., Buckberg G., Pradhan M., Roomi A.U., Gorissen W., Houle H. (2013). Two- and three-dimensional speckle tracking echocardiography: Clinical applications and future directions. Echocardiography.

[28] Chetboul V. (2010). Advanced techniques in echocardiography in small animals. Vet. Clin. N. Am.-Small Anim. Pract..

[29] Thavendiranathan P., Poulin F., Lim K.D., Plana J.C., Woo A., Marwick T.H. (2014). Use of myocardial strain imaging by echocardiography for the early detection of cardiotoxicity in patients during and after cancer chemotherapy: A systematic review. J. Am. Coll. Cardiol..

[30] Liu J.E., Barac A., Thavendiranathan P., Scherrer-Crosbie M. (2020). Strain Imaging in Cardio-Oncology. JACC CardioOncol..

[31] Acierno M.J., Brown S., Coleman A.E., Jepson R.E., Papich M., Stepien R.L., Syme H.M. (2018). ACVIM consensus statement: Guidelines for identification, evaluation and management of systemic hypertension in dogs and cats. J. Vet. Intern. Med..

[32] Chetboul V., Serres F., Gouni V., Tissier R., Pouchelon J.L. (2007). Radial strain and strain rate by two-dimensional speckle tracking echocardiography and the tissue velocity based technique in the dog. J. Vet. Cardiol..

[33] Wess G., Keller L.J.M., Klausnitzer M., Killich M., Hartmann K. (2011). Comparison of longitudinal myocardial tissue velocity, strain, and strain rate measured by two-dimensional speckle tracking and by color tissue Doppler imaging in healthy dogs. J. Vet. Cardiol..

[34] Amundsen B.H., Helle-Valle T., Edvardsen T., Torp H., Crosby J., Lyseggen E., Støylen A., Ihlen H., Lima J.A.C., Smiseth O.A. (2006). Noninvasive myocardial strain measurement by speckle tracking echocardiography: Validation against sonomicrometry and tagged magnetic resonance imaging. J. Am. Coll. Cardiol..

[35] Dandel M., Lehmkuhl H., Knosalla C., Suramelashvili N., Hetzer R. (2009). Strain and Strain Rate Imaging by Echocardiography—Basic Concepts and Clinical Applicability. Curr. Cardiol. Rev..

[36] Payne E.E., Roberts B.K., Schroeder N., Burk R.L., Schermerhorn T. (2011). Assessment of a point-of-care cardiac troponin I test to differentiate cardiac from noncardiac causes of respiratory distress in dogs. J. Vet. Emerg. Crit. Care.

[37] Siemann D.W., Chaplin D.J., Walicke P.A. (2013). A review and update of the current status of the Vasculature Disabling Agent Combretastatin-a4 phosphate (CA4P). Expert Opin. Investig. Drugs.

[38] Gould S., Westwood F.R., Curwen J.O., Ashton S.E., Roberts D.W., Lovick S.C., Ryan A.J. (2007). Effect of pretreatment with atenolol and nifedipine on ZD6126-induced cardiac toxicity in rats. J. Natl. Cancer Inst..

[39] Kalam K., Otahal P., Marwick T.H. (2014). Prognostic implications of global LV dysfunction: A systematic review and meta-analysis of global longitudinal strain and ejection fraction. Heart.

[40] Russo C., Jin Z., Elkind M.S.V., Rundek T., Homma S., Sacco R.L., Di Tullio M.R. (2014). Prevalence and Prognostic Value of Subclinical Left Ventricular Systolic Dysfunction by Global Longitudinal Strain in a Community-Based Cohort. Eur. J. Heart Fail..

[41] Collier P., Phelan D., Klein A. (2017). A Test in Context: Myocardial Strain Measured by Speckle-Tracking Echocardiography. J. Am. Coll. Cardiol..

[42] Cameli M., Mandoli G.E., Sciaccaluga C., Mondillo S. (2019). More than 10 years of speckle tracking echocardiography: Still a novel technique or a definite tool for clinical practice?. Echocardiography.

[43] Thavendiranathan P., Negishi T., Somerset E., Negishi K., Penicka M., Lemieux J., Aakhus S., Miyazaki S., Shirazi M., Galderisi M. (2021). Strain-Guided Management of Potentially Cardiotoxic Cancer Therapy. J. Am. Coll. Cardiol..

[44] Reant P., Labrousse L., Lafitte S., Bordachar P., Pillois X., Tariosse L., Bonoron-Adele S., Padois P., Deville C., Roudaut R. (2008). Experimental Validation of Circumferential, Longitudinal, and Radial 2-Dimensional Strain During Dobutamine Stress Echocardiography in Ischemic Conditions. J. Am. Coll. Cardiol..

[45] Steeds R.P. (2011). Echocardiography: Frontier imaging in cardiology. Br. J. Radiol..

[46] Negishi T., Thavendiranathan P., Negishi K., Marwick T.H., Aakhus S., Murbræch K., Massey R., Bansal M., Fukuda N., Hristova K. (2018). Rationale and Design of the Strain Surveillance of Chemotherapy for Improving Cardiovascular Outcomes: The SUCCOUR Trial. JACC Cardiovasc. Imaging.

[47] Surachetpong S.D., Teewasutrakul P., Rungsipipat A. (2016). Serial measurements of cardiac troponin I (cTnI) in dogs treated with doxorubicin. Jpn. J. Vet. Res..

[48] Lyon A.R., Dent S., Stanway S., Earl H., Brezden-Masley C., Cohen-Solal A., Tocchetti C.G., Moslehi J.J., Groarke J.D., Bergler-Klein J. (2020). Baseline cardiovascular risk assessment in cancer patients scheduled to receive cardiotoxic cancer therapies: A position statement and new risk assessment tools from the Cardio-Oncology Study Group of the Heart Failure Association of the European Society of Cardiology in collaboration with the International Cardio-Oncology Society. Eur. J. Heart Fail..

[49] Pudil R., Mueller C., Čelutkienė J., Henriksen P.A., Lenihan D., Dent S., Barac A., Stanway S., Moslehi J., Suter T.M. (2020). Role of serum biomarkers in cancer patients receiving cardiotoxic cancer therapies: A position statement from the Cardio-Oncology Study Group of the Heart Failure Association and the Cardio-Oncology Council of the European Society of Cardiology. Eur. J. Heart Fail..

[50] Luis S.A., Chan J., Pellikka P.A. (2019). Echocardiographic Assessment of Left Ventricular Systolic Function: An Overview of Contemporary Techniques, Including Speckle-Tracking Echocardiography. Mayo Clin. Proc..

[51] Kusunose K., Zhang Y., Mazgalev T.N., Thomas J.D., Popovic Z.B. (2013). Left ventricular strain distribution in healthy dogs and in dogs with tachycardia-induced dilated cardiomyopathy. Cardiovasc. Ultrasound.

[52] Suzuki R., Matsumoto H., Teshima T., Koyama H. (2013). Clinical assessment of systolic myocardial deformations in dogs with chronic mitral valve insufficiency using two-dimensional speckle-tracking echocardiography. J. Vet. Cardiol..

[53] Yingchoncharoen T., Agarwal S., Popović Z.B., Marwick T.H. (2013). Normal ranges of left ventricular strain: A meta-analysis. J. Am. Soc. Echocardiogr..

[54] Ünlü S., Mirea O., Bézy S., Duchenne J., Pagourelias E.D., Bogaert J., Thomas J.D., Badano L.P., Voigt J.U., Hamilton J. (2021). Inter-vendor variability in strain measurements depends on software rather than image characteristics. Int. J. Cardiovasc. Imaging.

[55] Yang H., Wright L., Negishi T., Negishi K., Liu J., Marwick T.H. (2018). Research to Practice: Assessment of Left Ventricular Global Longitudinal Strain for Surveillance of Cancer Chemotherapeutic-Related Cardiac Dysfunction. JACC Cardiovasc. Imaging.

[56] Suzuki R., Matsumoto H., Teshima T., Koyama H. (2013). Effect of age on myocardial function assessed by two-dimensional speckle-tracking echocardiography in healthy beagle dogs. J. Vet. Cardiol..

[57] Pedro B., Stephenson H., Linney C., Cripps P., Dukes-McEwan J. (2017). Assessment of left ventricular function in healthy Great Danes and in Great Danes with dilated cardiomyopathy using speckle tracking echocardiography. J. Vet. Cardiol..

[58] Westrup U., McEvoy F.J. (2013). Speckle tracking echocardiography in mature Irish Wolfhound dogs: Technical feasibility, measurement error and reference intervals. Acta Vet. Scand..

[59] Hernandez-Suarez D.F., Lopez-Menendez F., Roche-Lima A., Lopez-Candales A. (2019). Assessment of Mitral Annular Plane Systolic Excursion in Patients With Left Ventricular Diastolic Dysfunction. Cardiol. Res..

[60] Hu K., Liu D., Herrmann S., Niemann M., Gaudron P.D., Voelker W., Ertl G., Bijnens B., Weidemann F. (2013). Clinical implication of mitral annular plane systolic excursion for patients with cardiovascular disease. Eur. Heart J..

[61] Santarelli G., Talavera López J., Fernández del Palacio J. (2018). Evaluation of the right parasternal four-chamber view for the assessment of left ventricular longitudinal strain and strain rate by two-dimensional speckle tracking echocardiography in dogs. Res. Vet. Sci..

[62] Zois N.E., Tidholm A., Nägga K.M., Moesgaard S.G., Rasmussen C.E., Falk T., Häggström J., Pedersen H.D., Åblad B., Nilsen H.Y. (2012). Radial and Longitudinal Strain and Strain Rate Assessed by Speckle-Tracking Echocardiography in Dogs with Myxomatous Mitral Valve Disease. J. Vet. Intern. Med..

[63] Santarelli G., Baron Toaldo M., Bouvard J., Glaus T.M., Fernandez del Palacio J. (2019). Variability among strain variables derived from two-dimensional speckle tracking echocardiography in dogs by use of varioius software. Am. J. Vet. Res..

[64] Corda A., Pinna Parpaglia M.L., Sotgiu G., Zobba R., Gomez Ochoa P., Prieto Ramos J., French A. (2019). Use of 2-dimensional speckle-tracking echocardiography to assess left ventricular systolic function in dogs with systemic inflammatory response syndrome. J. Vet. Intern. Med..

[65] Baron Toaldo M., Guglielmini C., Diana A., Sarcinella F., Cipone M. (2014). Feasibility and reproducibility of echocardiographic assessment of regional left atrial deformation and synchrony by tissue doppler ultrasonographic imaging in healthy dogs. Am. J. Vet. Res..

[66] Abou R., Van Der Bijl P., Bax J.J., Delgado V. (2020). Global longitudinal strain: Clinical use and prognostic implications in contemporary practice. Heart.

[67] Johnson C., Kuyt K., Oxborough D., Stout M. (2019). Practical tips and tricks in measuring strain, strain rate and twist for the left and right ventricles. Echo Res. Pract..

